# The Role of Upper Body Biomechanics in Elite Racewalkers

**DOI:** 10.3389/fspor.2021.702743

**Published:** 2021-07-09

**Authors:** Helen J. Gravestock, Catherine B. Tucker, Brian Hanley

**Affiliations:** ^1^School of Health Sciences, Birmingham City University, Birmingham, United Kingdom; ^2^Carnegie School of Sport, Leeds Beckett University, Leeds, United Kingdom

**Keywords:** coaching, elite-standard athletes, endurance, kinematics, track and field

## Abstract

The aim of this study was to analyze the link between the upper and lower body during racewalking. Fifteen male and 16 female racewalkers were recorded in a laboratory as they racewalked at speeds equivalent to their 20-km personal records [men: 1:23:12 (±2:45); women: 1:34:18 (±5:15)]; a single representative trial was chosen from each athlete for analysis and averaged data analyzed. Spatial variables (e.g., stride length) were normalized to stature and referred to as ratios. None of the peak upper body joint angles were associated with speed (*p* < 0.05) and there were no correlations between pelvic motion and speed, but a medium relationship was observed between peak pelvic external rotation (right pelvis rotated backwards) and stride length ratio (*r* = 0.37). Greater peak shoulder extension was associated with lower stride frequencies (*r* = −0.47) and longer swing times (*r* = 0.41), whereas peak elbow flexion had medium associations with flight time (*r* = −0.44). Latissimus dorsi was the most active muscle at toe-off during peak shoulder flexion; by contrast, pectoralis major increased in activity just before initial contact, concurrent with peak shoulder extension. Consistent but relatively low rectus abdominis and external oblique activation was present throughout the stride, but increased in preparation for initial contact during late swing. The movements of the pelvic girdle were important for optimizing spatiotemporal variables, showing that this exaggerated movement allows for greater stride lengths. Racewalkers should note however that a larger range of shoulder swing movements was found to be associated with lower stride frequency, and smaller elbow angles with increased flight time, which could be indicative of faster walking but can also lead to visible loss of contact. Coaches should remember that racewalking is an endurance event and development of resistance to fatigue might be more important than strength development.

## Introduction

Racewalking is a technical event with its own unique gait pattern, determined by the athletes' attempts to maximize speed and to adhere to World Athletics Rule 54.2 (previously known as Rule 230.2). This rule states that racewalking is a progression of steps with no visible (to the human eye) loss of contact with the ground and that the athlete's advancing leg must be straightened from first contact with the ground until the vertical upright position (World Athletics, 2019). Although the upper body is not directly affected by Rule 54.2, it nonetheless functions to counterbalance the angular momentum of the lower body during racewalking (Hoga-Miura et al., 2016) and therefore the upper body might adopt particular movements to accommodate the rule. One major function of the arms' movements during normal walking and running is to counteract moments of the swinging legs around the vertical axis (Herr and Popovic, 2008; Pontzer et al., 2009), and this action is also considered by several coaches to be of particular importance in racewalking (Hopkins, 1981; Villa, 1990). Racewalking coaching literature has long recommended an emphasis on arm movements (e.g., considerable shoulder hyperextension at ipsilateral initial contact) (Payne and Payne, 1981; Drake, 2003), and indeed the vigorous arm swing in racewalking is accompanied by a transverse rotation of the thorax in an opposite direction to the pelvis, with thorax rotation in racewalking about double that of normal walking (Murray et al., 1983; Pavei and La Torre, 2016). Laboratory-based studies have found large transverse plane motions that generate torsion within the trunk (Pavei and La Torre, 2016) that coaches have long believed counteract the exaggerated movements of the pelvis (Hopkins, 1978), which themselves are emphasized to try to increase stride length (Knicker and Loch, 1990). However, whether the movements of the upper body, including the pelvis, have an effect on key spatiotemporal variables has not been fully established in racewalking to date.

Because the stance knee cannot flex until after the torso has passed over the foot, there is a profound effect on the contribution of the other joints during stance (Hanley and Bissas, 2017) as the lower limb becomes a rigid lever about which the upper body rotates, possibly affecting the path of the center of mass (CM). Similar to normal walking and running, it has been suggested that racewalkers improve mechanical efficiency by decreasing vertical displacement during walking (Murray et al., 1983). Cairns et al. (1986) reported the CM trajectory to be absent or diminished when racewalking because pelvic motions in the frontal plane (pelvic obliquity) reduce vertical CM movement and thereby increase efficiency. Because of pelvic obliquity, the hip is in the highest position over the center of the support leg and lowest when over the swinging leg (Pavei et al., 2014) and, in their small-scale study, Murray et al. (1983) noted the S-shape vertebral column reverses as weight is shifted between stance phases. This motion was proposed to help maintain a smooth vertical trajectory of the CM during racewalking (Murray et al., 1983; Cairns et al., 1986) under the old rules where the knee did not have to be straightened until the vertical upright position. More recently, Pavei et al. (2017) and Pavei et al. (2019) showed instead that the CM trajectory is closer to running than walking. The understanding of thorax motion in coaches would therefore benefit from further detailed analysis conducted in a laboratory using optoelectronics.

The magnitude of thorax torsion reported previously (Pavei and La Torre, 2016) suggests core abdominal muscles are important in racewalking, although the few studies of muscle activity using electromyography (EMG) in racewalking have focused on the lower limbs (Hanley and Bissas, 2013; Padulo et al., 2013; Cronin et al., 2016; Gomez-Ezeiza et al., 2019), with only one study of upper body muscle activity conducted before the current rule was introduced in 1995, and on a limited sample of two national-standard men (Murray et al., 1983). In competition, exaggerated upper body movements have indeed been found in kinematic studies of world-class racewalkers (Hanley et al., 2011, 2013), but the three-dimensional (3D) data analyzed were collected at 50 Hz and might not accurately describe movement in elite racewalking. Furthermore, these field-based studies used joint centers only to measure pelvic and thorax movements, and thus a novel 3D study using multiple markers and a higher sampling rate can provide more accurate information about how world-class racewalkers achieve their unique gait (Cazzola et al., 2016; Pavei and La Torre, 2016). In addition, the analysis of activity in key upper body muscles using EMG can explain their contribution to racewalking and provide a rationale for including specific training for these muscles (or muscle groups) within a strength and conditioning program.

Racewalk coaches have long emphasized arm movements and exaggerated pelvic and thoracic movements in trying to improve racewalking performances (Hopkins, 1981; Villa, 1990; Drake, 2003). However, the scientific rationale of these coaching recommendations for the upper body are not clearly established under the current rules for racewalking. Furthermore, it is not clear whether upper body movements influence racewalking speed and other key spatiotemporal measures that contribute to racewalking speed. Therefore, this study will make an original contribution to the literature by analyzing upper body kinematics with synchronized muscle activity patterns for both elite men and women racewalkers. Such information will impact coaches and athletes in informing training strategies and identify what kinematic movements are important for achieving high racewalking speeds and abiding by completion racewalking rules. Therefore, the aim of this study was to analyze the link between the upper and lower body movements during racewalking. This was achieved through a detailed biomechanical analysis on a large sample of elite standard men and women racewalkers using precise 3D motion capture and EMG to analyze the whole body during the entire gait cycle.

## Materials and Methods

### Research Approval

All human subjects were treated in accordance with established ethical standards. The protocol was approved by the Carnegie School of Sport Research Ethics Committee. All subjects gave written informed consent in accordance with the Declaration of Helsinki. The subjects were provided with Participant Information Sheets, and in accordance with the Carnegie School of Sport Research Ethics Committee's policies for use of human subjects in research, all subjects were informed of the benefits and possible risks associated with participation and informed of their right to withdraw at any time.

### Participants

Thirty-one racewalkers of 15 different nationalities volunteered for the study. Fifteen participants were men (age: 26 ± 5 years; stature: 1.78 ± 0.04 m; body mass: 64.7 ± 4.9 kg) and 16 were women (age: 28 ± 6 years; stature: 1.66 ± 0.08 m; body mass: 55.3 ± 9.4 kg). The sample included two IAAF World Championship medalists, a European Champion, a European Championship silver medalist, a World U20 Champion, a Commonwealth Games silver medalist, 13 other athletes who had competed at the Olympic Games and/or World Championships, two who had the Olympic qualifying time and three who had competed at the World U20 Championships or European Championships. The mean 20 km personal record time (h:min:s) for the men was 1:23:12 (±2:45), whereas for the women it was 1:34:18 (±5:15).

### Data Collection

**Figure 1:**
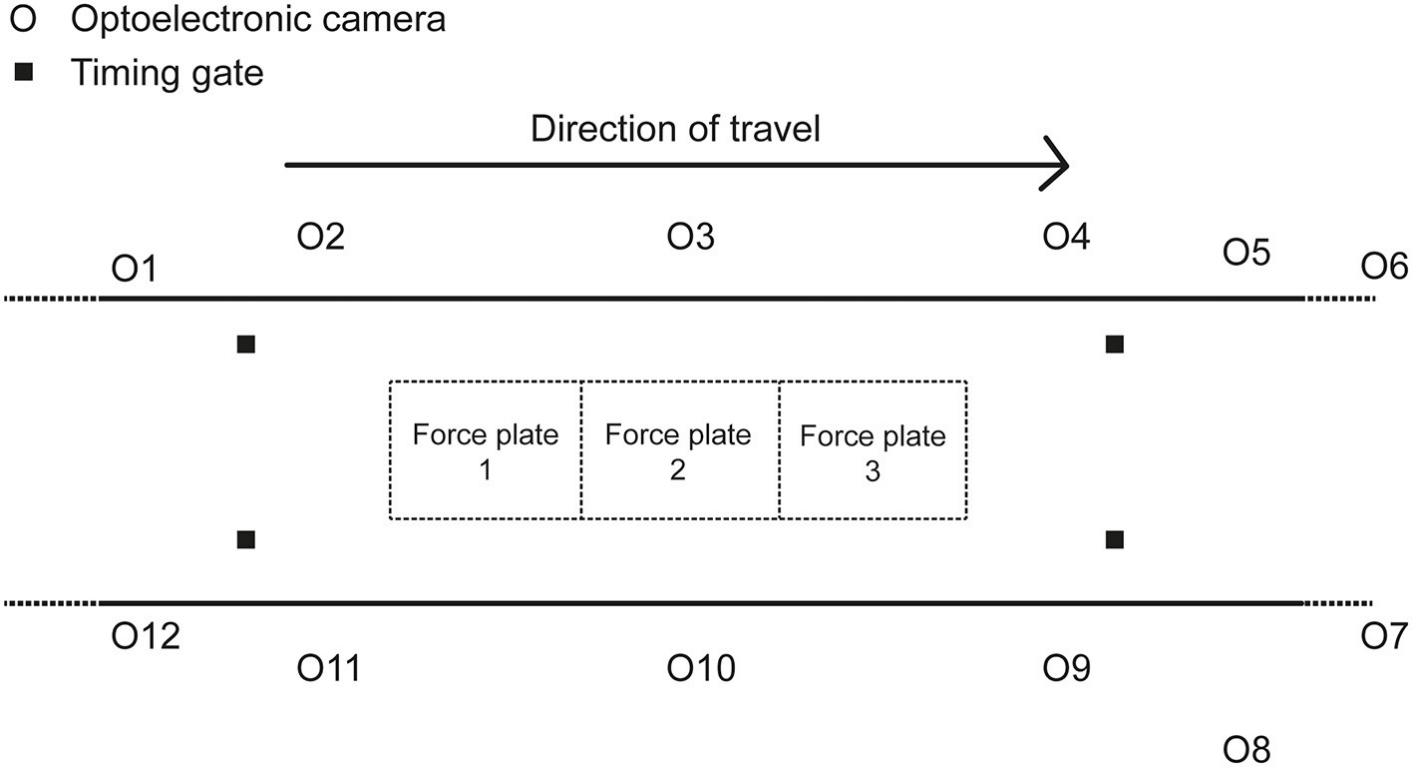
Diagram of the set-up of the data collection area in the biomechanics laboratory.

Each participant visited the laboratory on a single occasion. Participants wore tight fitting clothing and their usual training shoes. Having performed several familiarization trials before testing, the participants racewalked along a 45-m indoor track in the biomechanics laboratory at race pace (Figure 1); a minimum of six acceptable trials were recorded, defined as the athlete achieving the target speed (±5%), clean foot contacts on at least one force plate, and with no visible evidence of targeting or breaching of racewalk competition rules (one of the study authors, who was present during all data collection, is a qualified racewalking judge). There was at least a 30-s rest period between trials.

Each trial involved the participants racewalking multiple times across three successively positioned force plates (9287BA, Kistler Instruments Ltd., Winterthur). The force plates sampled at 1000 Hz and were 900 mm long and 600 mm wide [natural frequency ≈ 750 Hz (x-, y-), ≈ 520 Hz (z-); linearity < ±0.5% full scale output; cross talk < ±1.5%; hysteresis <0.5% full scale output]. The force plates were covered with a synthetic athletic surface to make them flush with the rest of the track and ensure ecological validity (Bezodis et al., 2008). Force plate voltages were recorded through Qualisys Track Manager Software (QTM) (v2.17, Qualisys, Gothenburg). Each participant's racewalking pace was recorded using double photocell timing gates (Microgate, Witty, Bolzano) placed 4 m apart and positioned around the force plates. Kinematic data were simultaneously recorded using a 12-camera optoelectronic motion analysis system (Oqus 7, Qualisys, Gothenburg) operating at 250 Hz. A configuration of 70 reflective markers and marker clusters were placed on anatomical landmarks and segments to describe participants' segment kinematics (Table 1). The capture volume was calibrated according to the manufacturer's instructions. Given that a large capture volume was used, an error of <2 mm for the dynamic calibration was accepted. The mean calibration error from all testing sessions was 1.45 ± 0.23 mm.

**Table 1:** Description of the segments used for kinematic analysis.

**Segment**	**Description**
Head	Bilateral markers on front and back of head
Thorax	Bilateral markers on acromion process, C7 spinous process, T7 spinous process, suprasternal notch, xiphoid process
Pelvis	Bilateral markers on ASIS and PSIS
Right and left upper arm	Acromion process, three-marker tracking cluster, humeral lateral, and medial epicondyles
Right and left forearm	Humeral lateral and medial epicondyles, three-marker tracking cluster, radial and ulnar styloid processes
Right and left hand	Radial and ulnar styloid processes, marker on center of dorsal aspect of hand
Right and left thigh	Greater trochanter, medial and lateral femoral epicondyles, four-marker tracking cluster
Right and left shank	Medial and lateral femoral epicondyles, four-marker tracking cluster, medial and lateral malleoli
Right and left foot	Medial and lateral malleoli, first and second metatarsal heads
Right and left forefoot	Calcaneus, first and fifth metatarsal heads

Surface EMG was recorded with eight wireless sensors (Delsys Trigno, Delsys Inc, USA), sampling at 1926 Hz. The EMG sensors had four silver 5 × 1 mm bar contacts with an inter-electrode distance of 10 mm. Raw signals were measured at a bandwidth of 20–450 Hz using a differential amplifier. The common mode rejection ratio (CMRR) was ≤−80 dB at 60 Hz and input impedance was <10 Ω. Baseline noise was <750 μV root mean square (RMS), and effective signal gain was 909 V/V ± 5%. The EMG sensors were attached to the right side only and were positioned in the direction of the muscle fibers (Clarys and Cabri, 1993). Before placing the EMG sensors, the skin surface was prepared by shaving (if necessary), lightly abrading and cleaning with an isopropyl alcohol swab. EMG sensors were placed on the biceps brachii, middle deltoid, pectoralis major, latissimus dorsi, trapezius middle fibers, trapezius lower fibers, rectus abdominis and external oblique. These muscles were selected based on previous research that analyzed upper body muscle contributions in normal walking (Goudriaan et al., 2014). The EMG sensors were positioned in line with the Surface Electromyography for the Non-Invasive Assessment of Muscles (SENIAM) guidelines (Hermens et al., 2000). Simultaneous data capture of all systems was initiated using a trigger module (Delsys Inc., USA). Data from the optoelectronic system and force plates were recorded through QTM (v. 2.17, Qualisys, Gothenburg). EMG data were stored in EMGworks Acquisition software for post-processing.

### Data Analysis

3D kinematic marker trajectories from the motion trials were labeled from which Automatic Identification of Markers (AIM) models were generated (Qualisys, Gothenburg) (Rodger et al., 2013). Gap filling was completed when fewer than 10 consecutive frames were missing in the trajectory. Gait events were identified using the force plate data (O'Connor et al., 2007; Zeni et al., 2008); the vertical component of the ground reaction force (GRF) was used to determine the timing of each gait event. The instant of initial contact occurred when the vertical GRF exceeded three standard deviations (SD) above force plate noise (Addison and Lieberman, 2015), which itself was calculated from the first 50 samples of unloaded data for each trial. The mean threshold found using this method was 5 N (±5). Where gait events occurred without force plate contact, the vertical velocity of the calcaneus marker was used. The vertical velocity of this marker was found to have a characteristic shape, repeated in each gait cycle. By locating the relevant vertical velocity value of the heel marker at the time instant defined by vertical GRF data, the subsequent gait event could be identified using this velocity value validated from the gold standard event identification method. A similar principle was used by O'Connor et al. (2007) who used foot CM velocity in an algorithm to identify gait events.

Kinematic data were exported to Visual3D (V6 x64, C-Motion Inc., Maryland) using a custom-made Project Automation Framework (Qualisys, Gothenburg). Data from the static and tracking markers in a standing trial were used to develop a six degrees of freedom (6DOF) 3D whole-body kinematic model in Visual3D. All body segment parameter values were estimated from total body mass using regression analysis conducted by Dempster (1955). The center of gravity location and moments of inertia for each segment was calculated using the Hanavan (1964) mathematical model, an approach that has been validated through comparisons using experimental data (Hanavan, 1964). Shoulder, elbow, thorax, pelvis, hip, knee, ankle, ankle, and forefoot angles were calculated based on the Cardan angle rotation order of XYZ. Kinematic data were filtered using a fourth-order zero-lag low pass Butterworth digital filter. Residual analysis was carried out to determine the optimal cut-off frequency (Winter, 2005); the results showed an optimal cut-off frequency ranging from 11.1 to 12.8 Hz so it was decided to use 12 Hz as the cut-off frequency.

EMG data were filtered using the in-built hardware bandpass filter (20–450 Hz). Signal bandwidth was 430 Hz where the slopes of the lower (20 ± 5 Hz) and upper (450 ± 50 Hz) cut-offs were >40 dB/dec and >80 dB/dec, respectively. EMG data were processed in EMGworks analysis software (v.4.2.7 Analysis, Delsys, Massachusetts). To remove the effects of any offset, the mean was removed from each raw EMG reading for each of the eight muscles (Chuang and Acker, 2019). Data were cropped in the time domain to the same gait events as the kinematic data. An RMS calculation was applied with a moving window size of 50 ms and overlap of 25 ms with zero padding (Hanley and Bissas, 2013). A cubic spline was used to smooth and interpolate the data to 101 points. Similar to the kinematic data, the stance phase data were interpolated to 51 points, and the swing phase data interpolated to 50 points to ensure toe-off visually occurred at 50% of the gait cycle. This process was adopted to visualize the two different phases in the right leg, rather than to state categorically the relative duration of each phase; note that flight time occurs when both legs are in swing: one in early swing, and the other in late swing, and will therefore occur near the start and end of each cycle.

For each participant, at least six good trials were collected at race pace (i.e., equivalent to their season's best performance), and one successful trial was selected for analysis. A successful trial was defined using the same criteria during data collection with the additional conditions of good tracking data with minimal marker drop out, and one whole gait cycle (i.e., one single stride) for the right side recorded within the capture volume. Spatiotemporal variables were computed using the initial contact and toe-off gait events. Both spatiotemporal and joint angle variables were calculated using Visual3D. Stride length was defined as the perpendicular anteroposterior distance between subsequent right foot initial contacts. Stride length was also expressed as a percentage of the participants' statures, as this is the easiest method of normalization for coaches given difficulties in accurately measuring leg length, and referred to as stride length ratio. Foot ahead and foot behind distances were defined as the anteroposterior distances between the whole body CM and the foot segment CM at initial contact and toe-off, respectively. Flight distance, which is a component of stride length, was defined as the anteroposterior travel distance of the whole body CM during any flight phase (Hunter et al., 2004). Stride width was defined as the mediolateral distance from the proximal end position of the foot (the toes) at initial contact to the toes of the contralateral foot at the subsequent initial contact. As with stride length, these distances were also expressed as a proportion of stature, and referred to as ratios. Stance time and swing time were calculated as the time between right initial contact to right toe-off and right toe-off to right initial contact, respectively. Flight time was calculated as the time between right foot toe-off and left foot initial contact. Stride frequency (Hz) was calculated as the reciprocal of stride time.

Joint angle maxima and minima were calculated over the gait cycle with the timings of these events determined. Joint angle time series data have been presented with the percentage of gait cycle for each of the major upper and lower body joints for the predominant planes of motion. An additional angle, the forefoot angle, was calculated at the foot to monitor the sagittal plane angle at the metatarsophalangeal joints. This angle was included as during the contact phase the foot rolls forward around the forefoot leaving the tip of the toe as the last contact point. Movement of the forefoot toward the tibia in the sagittal plane was considered plantarflexion. Pelvic and thorax internal rotation described rotation of the right side of the body forwards (in the direction of hip flexion), whereas external rotation described rotation of the right side backwards (in the direction of hip hyperextension).

### Statistics

Results are reported as means ±1 SD. Density ridgeline plots display the relative timing of key joint actions during the gait cycle and were interpreted in a similar way to a smoothed histogram. In effect, ridgeline plots are partially overlapping line plots that resemble a mountain range, and are considered useful for visualizing changes in distributions over time (Wilke, 2017). The height of each curve represents the frequency of each event in the gait cycle (%); the width of each curve along the x-axis denoted the time window at which the joint reached maximum or minimum values. For the smoothed appearance, a kernel density estimate was used to produce the shaded probability density functions. This estimate was based on the probability density function of each variable using a 3.6 bandwidth. Density ridgeline plots were produced in R (Version 1.1463, Rstudio Inc., Boston, MA) using ggridges library (Wilke, 2017). Spatiotemporal group means with associated SD were also calculated. Pearson's product moment correlation coefficient (*r*) was used to find associations between spatiotemporal, minimum, and maximum joint angle data. A confidence level of 5% was set. Significant correlations were considered to be small (*r* = 0.10–0.29), medium (*r* = 0.30–0.49) or large (*r* ≥ 0.50) (Cohen, 1992). All statistical analyses were conducted using SPSS Statistics 19, Release Version 25.0.0 (IBM SPSS, Inc., 2010, Chicago, IL). EMG data were presented with appropriate joint kinematics with respect to the gait cycle; this involved finding the maximum value for each individual (in V) and expressing each value in the sequence as a percentage of the maximum. These percentage data were then averaged across all participants.

## Results

None of the normalized (ratio) variables differed between sexes, and consequently the means, SDs and ranges for men and women have been combined for the purposes of description and analysis and are presented in Table 2. To put some of the results below into context, notable significant associations were found between speed and stride length ratio (*r* = 0.65) and stride frequency (*r* = 0.67), between flight time and flight distance ratio (*r* = 0.71), and between flight time and stride length ratio (*r* = 0.52); it should be noted that flight distance will contribute to overall stride length, but also that this individual component of stride length is important in racewalking speed.

**Table 2:** Group mean ± SD data for spatiotemporal variables for men, women, and all athletes (combined data).

**Variable**	**Men**	**Women**	**All athletes**
Speed (m/s)	3.99 ± 0.29	3.60 ± 0.35	3.79 ± 0.38
Stride length (m)	2.41 ± 0.14	2.22 ± 0.14	2.31 ± 0.17
Stride length ratio (%)	136 ± 7.0	134 ± 6.3	135 ± 7
Stride frequency (Hz)	1.65 ± 0.08	1.63 ± 0.15	1.64 ± 0.12
Foot ahead (m)	0.36 ± 0.03	0.33 ± 0.03	0.34 ± 0.03
Foot ahead ratio (%)	19.9 ± 1.0	19.6 ± 1.6	19.7 ± 1.4
Foot behind (m)	0.47 ± 0.03	0.44 ± 0.04	0.45 ± 0.04
Foot behind ratio (%)	26.5 ± 2.0	26.3 ± 1.7	26.4 ± 1.7
Flight distance (m)	0.21 ± 0.05	0.18 ± 0.06	0.19 ± 0.05
Flight distance ratio (%)	11.8 ± 2.6	10.9 ± 3.4	11.3 ± 3.4
Flight time (s)	0.049 ± 0.010	0.048 ± 0.020	0.048 ± 0.016
Stance time (s)	0.254 ± 0.020	0.266 ± 0.041	0.260 ± 0.032
Swing time (s)	0.351 ± 0.015	0.354 ± 0.027	0.353 ± 0.021
Stride width (m)	0.05 ± 0.03	0.05 ± 0.02	0.05 ± 0.03

**Figure 2:**
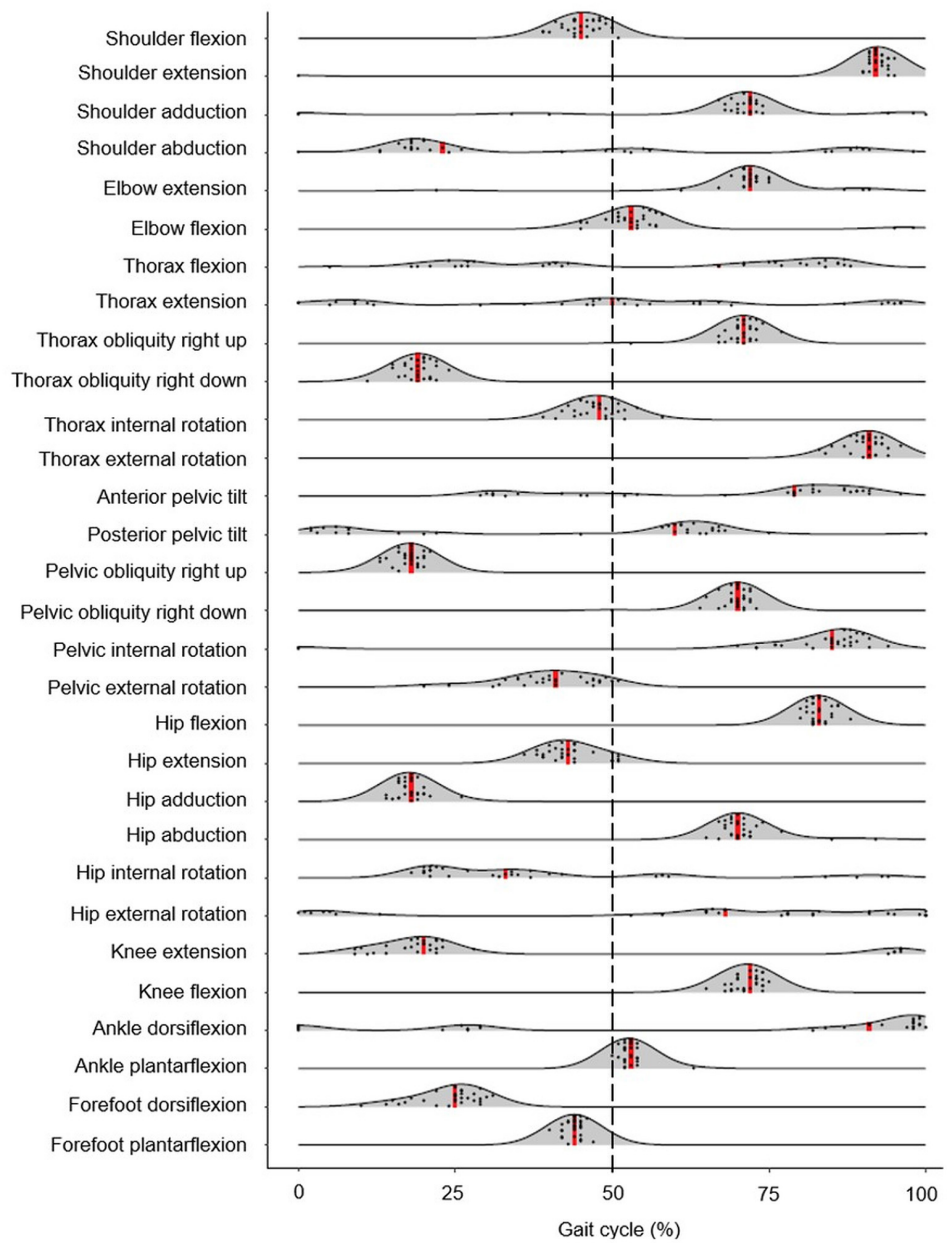
Density ridgeline plot of the timing of each joint angle peak during the gait cycle. Individual variation is dotted, and median values are represented by red vertical lines. The dashed vertical line represents gait cycle division between stance (from 0 to 50%) and swing (from 51 to 100%).

**Figure 3:**
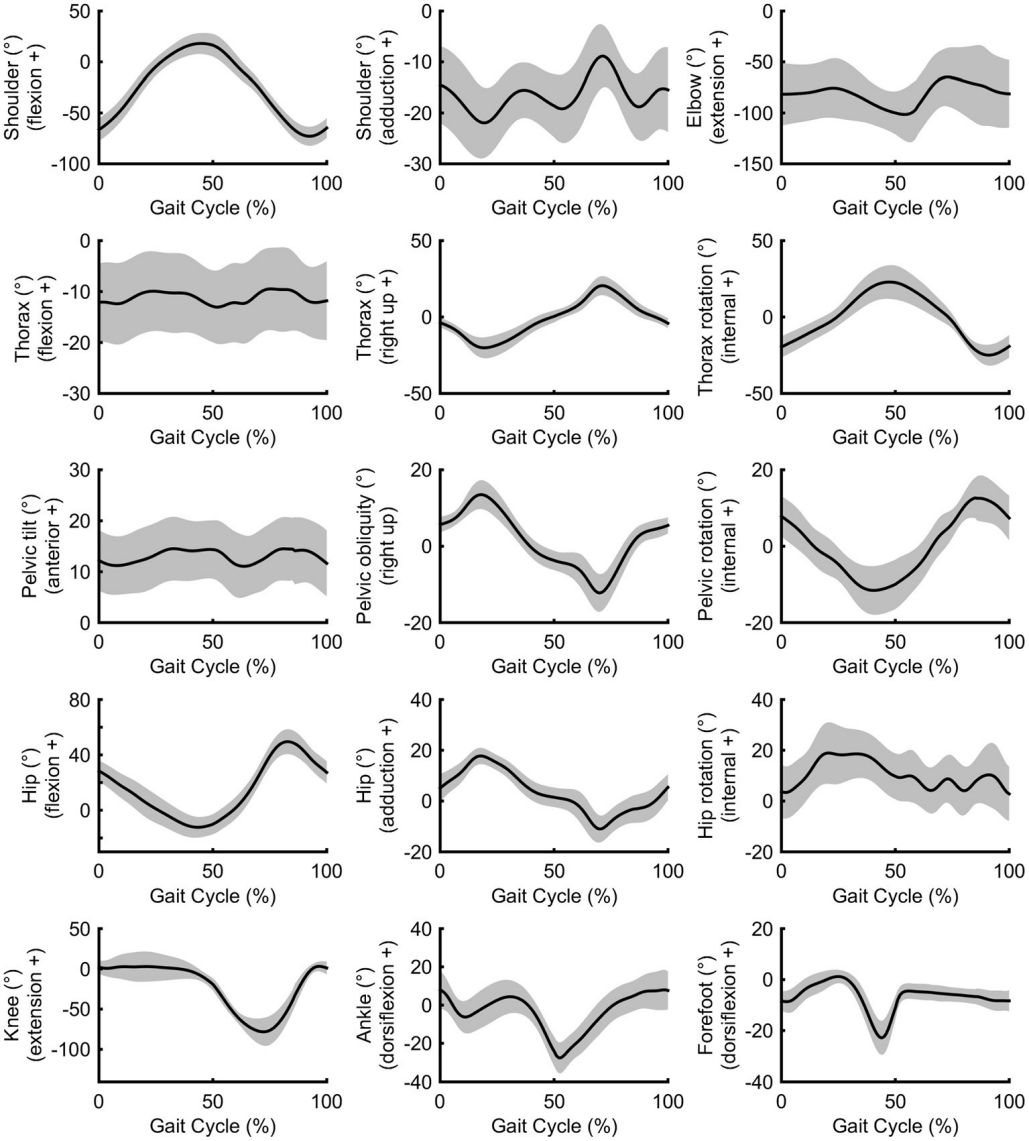
Group mean (± SD) upper and lower body joint angle kinematics.

Peak shoulder flexion occurred just before ipsilateral toe-off, approximately at the same time as peak thorax internal rotation, pelvic external rotation, hip extension, and forefoot plantarflexion (Figure 2). Peak movements in the opposite direction (shoulder extension, thorax external rotation, pelvic internal rotation) occurred just before ipsilateral initial contact during late swing; greater peak shoulder extension was associated with lower stride frequencies and longer swing times, whereas peak shoulder flexion was correlated with foot behind ratio (Table 3). Peak shoulder adduction occurred during early swing to assist its extension movement; similarly, peak shoulder abduction occurred during shoulder flexion (Figures 2, 3). Forefoot dorsiflexion occurred at midstance, at roughly the same time as peak knee extension (or hyperextension), hip adduction, pelvic obliquity (right side up) and thorax obliquity (right side down) (Figure 2). Their opposing movements (knee flexion, hip abduction, pelvic obliquity (right side down) and thorax obliquity (right side up) occurred during midswing. Peak ankle plantarflexion occurred immediately after toe-off, although peak ankle dorsiflexion varied more within the group (Figure 2); for most athletes it occurred just before initial contact (Figure 3). The timing of many other kinematic measures, such as anterior and posterior pelvic tilt, hip rotation and thorax flexion/extension, was also more varied across the group of athletes, as shown by the spread across the x-axis in Figure 2 and by the SD clouds in Figure 3. None of the peak upper body joint positions were associated with speed, although pelvic external rotation was negatively correlated with stride length ratio and foot behind ratio (Table 3), and stride width was positively correlated with pelvic internal rotation, hip abduction and hip internal rotation and negatively with hip external rotation.

**Table 3:** Relationships (*r*) between spatiotemporal variables and upper body minimum and maximum joint angles.

**Peak joint position**	**Stride length ratio**	**Stride frequency**	**Foot behind ratio**	**Stance time**	**Swing time**	**Stride width**
Shoulder extension	0.01	−0.47^**^	−0.05	0.32	0.41^*^	0.20
Shoulder flexion	0.30	0.06	0.40^*^	−0.01	−0.10	0.05
Pelvic obliquity right down	−0.02	−0.26	0.39^*^	0.33	0.07	0.24
Pelvic obliquity right up	−0.06	−0.32	0.31	0.39^*^	0.13	0.22
Pelvic external rotation	0.37^*^	−0.16	0.44^*^	0.09	0.11	0.07
Pelvic internal rotation	−0.11	0.05	0.06	0.17	−0.29	0.38^*^
Hip adduction	0.10	0.05	−0.06	−0.16	0.05	−0.30
Hip abduction	−0.01	−0.30	0.37^*^	0.39^*^	0.06	0.53^**^
Hip external rotation	0.32	−0.19	0.30	0.09	0.23	0.45^*^
Hip internal rotation	0.18	−0.35	0.35	0.26	0.31	0.49^**^

*Correlations were significant at p < 0.05*

(^*^) and p < 0.01

(^**^). All values are presented as magnitudes (no associated direction) to make interpretation simpler.

**Figure 4:**
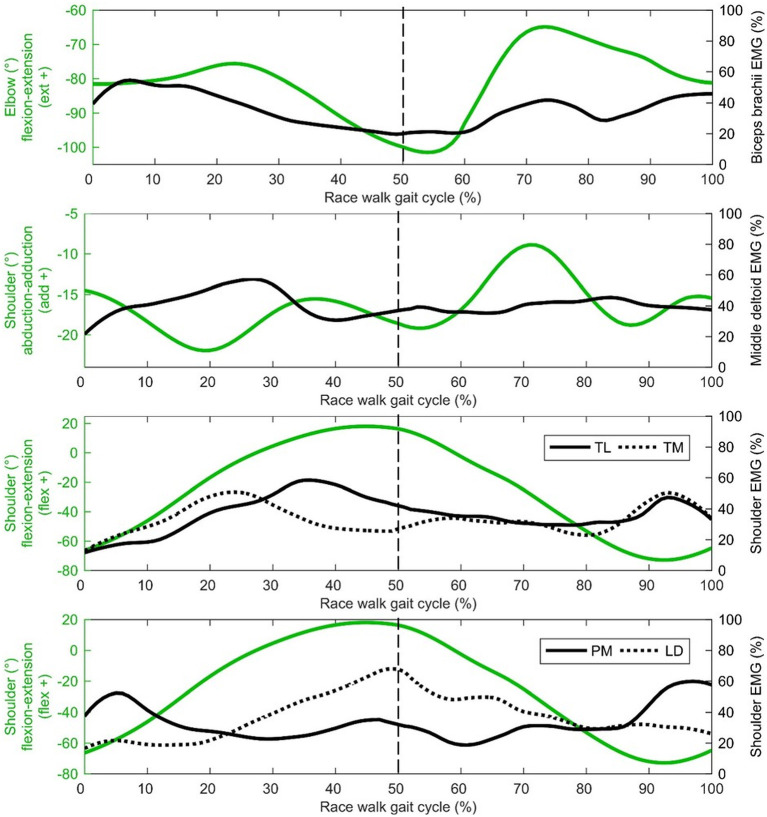
Shoulder and elbow group mean joint angle kinematics (green) presented with EMG activity (black) on the secondary axis [biceps brachii, middle deltoid, trapezius lower (TL), trapezius middle (TM dotted), pectoralis major (PM), latissimus dorsi (LM dotted)]. The dashed vertical line represents gait cycle division between stance (from 0 to 50%) and swing (from 50 to 100%).

Regarding the shoulder muscles, latissimus dorsi was the most active muscle at toe-off (Figure 4) when the shoulder was at peak flexion. By contrast, the pectoralis major trace in Figure 4 shows an increase in activity just before and after initial contact, when the shoulder was at peak extension. Biceps brachii, also an elbow muscle, shows a bimodal pattern of two peaks that occur during the first part of the stance phase and terminal swing phase, when the elbow was flexed least (Figure 4). These periods of the gait cycle fall just after and before initial contact, where the shoulder flexion-extension moves out of phase with hip flexion-extension (Figure 3). The elbow was found to be most flexed near toe-off when the shoulder is also in peak flexion, positioned in front of the body (Figure 3). Peak elbow flexion was correlated with flight time (*r* = −0.44). With regard to the trapezius muscle, the middle fibers peak near the middle of the stance phase (~23% of gait cycle) and the lower fibers peak near terminal stance and toe-off (~36% of gait cycle) (Figure 4).

**Figure 5:**
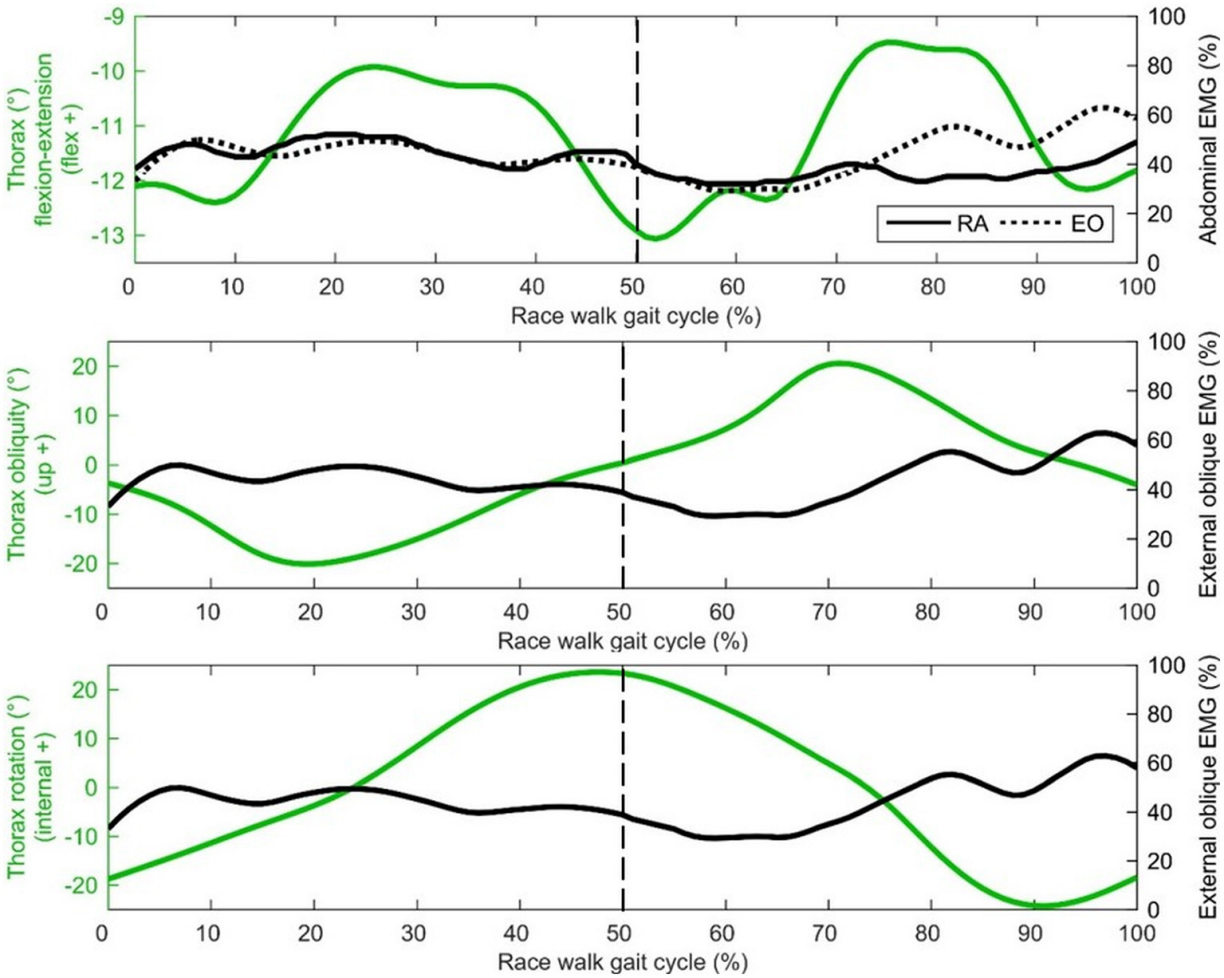
Thorax group mean joint angle kinematics (green) presented with EMG activity (black) on the secondary axis [rectus abdominis (RA), external oblique (EO dotted)]. The dashed vertical line represents gait cycle division between stance (from 0 to 50%) and swing (from 50 to 100%).

Middle deltoid muscle activation shows one burst of activity at about 30% of the gait cycle (Figure 4), after the shoulder has returned from its most abducted position in the gait cycle and adducts to a more neutral position that is maintained for most of the gait cycle (Figure 3). Rectus abdominis has been presented with thorax flexion-extension in Figure 5, which shows muscle activity patterns of the upper body during the gait cycle. Both thorax flexion and extension had no clear peak that was consistent across the group of athletes (Figure 2), most likely because the angle changed little during the stride (Figure 3). The activation magnitude of rectus abdominis fluctuates between 32 and 52% of the gait cycle. Slightly increased rectus abdominis activity occurs at 20%, 40%, and then again between 70 and 90% of the gait cycle. Similar to rectus abdominis, consistent external oblique activation is present throughout the gait cycle that is relatively low but increased in preparation for initial contact during terminal swing (Figure 5).

## Discussion

The aim of this study was to analyze the link between the upper and lower body movements during racewalking. The inclusion of spatiotemporal variables is important given that there is a specific rule defining the motion, and therefore any interpretation of the role of upper body movement should take these into account. Flight time is of particular interest to racewalkers and coaches given that World Athletics Rule 54.2 states that no visible loss of contact should occur. Each participant incurred a flight phase to some extent, and as the group's mean flight time was 0.048 s, the racewalkers in this study were thus individually below or only just above the detectable threshold of 0.045 s (Hanley et al., 2019). Longer flight times were associated with stride length ratio and flight distance ratio, which in normal running would be of benefit but in racewalking must be restrained to avoid detection by the judges. We should note that flight distance is not separate from stride length, but one component of it, and so a reduction or absence of flight distance will reduce stride length and, most likely, speed. Because the racewalker cannot use a long flight phase to increase stride length, other movements are exaggerated to try to achieve this (Pavei and La Torre, 2016). For example, racewalkers use a greater range of pelvic rotation about the vertical axis than normal walking (Cairns et al., 1986). This theoretically helps the racewalker maintain a narrow step width to help increase step length (Murray et al., 1983), and although there were no correlations between pelvic motion and speed, a positive relationship was observed between peak pelvic external rotation and stride length ratio showing that this movement does achieve this goal in practice (Pavei and La Torre, 2016), and should continue to be encouraged by coaches. Peak external pelvic rotation occurs at toe-off, and therefore increases the foot behind distance component of stride length, and occurs at the same time as the ipsilateral shoulder's peak flexion position (in front of the body), showing that this movement helps to counterbalance the lower limb's movement (Pavei and La Torre, 2016).

In terms of shoulder musculature, peak pectoralis major activation occurred as the shoulder moved to its most hyperextended behind the body at initial contact, whereas latissimus dorsi activity peaked at toe-off, occurring as the shoulder and elbow reach peak flexion. Although we did not measure joint powers because we did not have GRF data for all strides, it is probable that these muscles were absorbing energy during these respective phases and effectively working to decelerate backward and forward arm swing in preparation for the reverse movement. The role of these muscles might therefore be one of elastic energy storage and return, and a more forced arm swing that has been recommended by coaches (e.g., Payne and Payne, 1981) does not appear to occur in practice; indeed, given that racewalking is an endurance event, it could be more important that racewalkers develop upper body musculature fatigue resistance (as in the lower limb) to maintain correct technique. Research has shown that the middle deltoid and supraspinatus have nearly equal cross sectional areas and moment arms for shoulder abduction (within 10–12% of each other), and each muscle produces a roughly equal share of the total abduction torque at the shoulder joint (Neumann, 2017). It could be that the supraspinatus had greater muscle activation during racewalking but, as it is located deep to the trapezius, is not suitable for surface EMG measurement. Nonetheless, it is clear that the shoulder muscles (acting in the sagittal and frontal planes) have important roles in racewalking to achieve fast movements, and the development of an optimal range of movement (with requisite strength and conditioning training) that balances well the lower body is recommended. This might be particularly important with regard to restraining shoulder hyperextension during late swing given larger magnitudes were associated with reduced stride frequencies.

The middle deltoid muscle was most active during midstance as the shoulder was abducted during the forward arm swing. The middle and lower trapezius muscles were also at their most active during this phase, possibly as they function to retract and depress the shoulder girdle, corresponding with peak thorax obliquity (right down) and pelvic obliquity (right up); this combination of movements contributes to the S-shape of the vertebral column during stance that is a hallmark of racewalking (Murray et al., 1983). With regard to the counterbalancing movements of the thorax, the consistently low activation profile of the rectus abdominis is suggestive of a constant isometric action to maintain upper body posture throughout the racewalking gait cycle. It was noticeable from Figures 2, 3 that for many movements, there was no consistent pattern for all athletes, which was most likely because the range of motion was quite small (and peak magnitudes were therefore not that discernable). The similar lack of large peak values for the EMG data (i.e., few values close to 100%) was indicative of how athletes tended to attain peak muscle activity values at different phases of the movement. Other authors have noted very low and variable activity throughout the normal walking gait cycle (Cromwell et al., 2001; Anders et al., 2007). Previous research found no rectus abdominis activity or no clear relationship with lumbo-pelvic motion during normal walking (Mann et al., 1986; Callaghan et al., 1999), but high activity in association with initial contact in running (Mann et al., 1986). When comparing variables such as duty factor, CM energy and vertical GRF magnitude, racewalking is closer to running (Cavagna and Franzetti, 1981; Pavei et al., 2014, 2017, 2019) than to normal walking, but nonetheless the thorax kinematics in our study of racewalkers were not enough to elicit the muscle activity magnitudes observed in running (Pontzer et al., 2009). In studies where rectus abdominis muscle activity has been monitored alongside hip flexor activation, it has been noted that activity of these muscle sites is somewhat synchronized (Neumann, 2017). At slower walking speeds, Anders et al. (2007) noted similar continuous activation profiles between rectus abdominis and the external obliques, but at faster speeds a mixed phasic activation pattern was recorded.

In the present study, slightly greater external oblique activation occurred during early stance phase and terminal swing, similar to the patterns presented by Anders et al. (2007), and the rectus abdominis activation pattern noted above. With respect to thorax kinematics, the external oblique muscle activation pattern appears to follow rotation in the transverse plane, with greater activity observed with external thorax rotation. Therefore, external oblique activity is mostly isometric, with eccentric muscle actions when the thorax is high and externally rotated. Murray et al. (1983) noted increased activity of the lateral abdominal muscles was related to reversals of the extremes of the lateral trunk flexion associated with racewalking. Furthermore, the increased activity of the abdominal muscles could limit the amount of pelvic tilt, which was found to be one of the few kinematics not in excess of normal walking when racewalking (Murray et al., 1983), and which did not have a distinct timing for its peak across all athletes in the present study. Greater external oblique activation at terminal swing has also been noted in response to perturbations during normal walking (Stokes et al., 2017). From a coaching perspective, Drake (2003) suggested that a lack of core stability results in sub-optimal hip flexion-extension and is a contributing factor to illegal knee motion or visible flight phase technical errors, although our data were not conclusive in showing whether this was the case; there were no correlations between thorax movements and flight time, although the sample studied were homogenous in that they were elite athletes and typically engaged in abdominal strength exercises.

Given the relatively low percentage of muscle activation from the biceps brachii throughout the gait cycle, it would appear that little muscular action produces the cyclical flexion-extension pattern at the elbow and instead this could largely be a passive motion resulting from the angular momentum of the lower body (somewhat similar to the shoulder), albeit with some muscular work needed to overcome friction. Furthermore, biceps brachii activity is lowest when elbow flexion is greatest. It could be that the role of the biceps brachii is to maintain elbow flexion throughout the arm swing that might be compromised because of gravity, especially on the downswing where a noticeable burst of activation occurs. This activation begins as the elbow is extending relatively quickly compared with the rest of the elbow flexion-extension trace. An energy absorbing action is therefore performed by the biceps brachii to initiate the burst in activity just after 60% of the gait cycle. This action then changes to isometric, whereas elbow flexion angle remains relatively constant in preparation for initial contact between 90 and 100% of the gait cycle. This looks to continue after initial contact where muscle activation and elbow flexion is sustained from initial contact to ~25% of the gait cycle, which could be to flex the shoulder as Murray et al. (1983) also showed a burst in biceps brachii activity immediately after initial contact that they believed initiated the forward thrust of the arm swing. Indeed, the lack of considerable differences in EMG descriptions between the present study and that of Murray et al. (1983) is indicative of how little the racewalking rule change (in 1995) affected muscle activity in the upper body. Racewalkers with smaller elbow angles in early swing when the elbow was most flexed had longer flight times, which can be beneficial for speed, but also lead to detectable flight times and maintaining a more extended position, and therefore greater arm moment of inertia, might be better. However, the mass of the forearm is so small, and as the movement of one arm is counterbalanced by the opposite movement of the contralateral arm, it is unlikely that it is the arm movement itself that causes these longer flight times; rather, the arm's movement might be symptomatic of other movements causing these actions. The arms' movements, therefore, might be a reaction rather than a cause. Indeed, the data showed that the athletes' elbows through flexion-extension while racewalking (Pavei and La Torre, 2016), rather than maintaining a set angle (e.g., of 90°) as recommended by coaches (Markham, 1989; Rogers, 2000), and this variance might help racewalkers reduce the duration of any flight phase (which often occurs during early swing, when the elbow was most flexed).

With regard to potential limitations, it was assumed that elite racewalkers would be symmetrical as their well-trained gait has been found to show low asymmetry for the most important kinematic variables across racewalking speeds (Tucker and Hanley, 2020). All EMG data in the present study were collected for the right side only, in line with other studies that have measured muscle activity of the right lower limb in elite racewalkers (Hanley and Bissas, 2013) and normal walking and running (Saunders et al., 2005). An alternative approach might have been to pool left and right side data together. Where this method has been used, a common limitation was acknowledged in that pooling data smooths out individual participation and that these overall means cannot be perceived as a true profile of muscle activity. From a practical perspective, the number of EMG sensors available was limited to eight, and so increasing the number and different functions of muscles tested was prioritized over bilateral analysis. It is also accepted that analysis of movement in the laboratory environment might not be representative of racewalking in competition. It was also decided in this study to use the maximum EMG value within each muscle for each individual as a basis to normalize their EMG data before averaging across the group; this might have led to difficulties in establishing distinct patterns of activation. An alternative approach could have been to normalize relative to maximum voluntary contraction (MVC), but this was considered too difficult to achieve for many of the muscles analyzed given the multiplanar movements of the joints they cross. However, the value of synchronized EMG and 3D motion capture analyses to coaches and athletes was considered to outweigh the ecological validity of competition data and provide an original contribution to the knowledge base of racewalking biomechanics. Furthermore, the laboratory approach of testing each participant individually one at a time meant that greater sample sizes could be included in the analysis to better represent the movements of world-class racewalkers. We included only one stride per participant (rather than the mean of the six good trials collected) to ensure that the trial included was not affected by the averaging process across multiple trials. However, this means the total number of strides analyzed was limited to 31 (one for each participant) and this might restrict the generalizability of our results.

## Conclusions

This was the first study to analyze the effect of upper body movements on spatiotemporal variables and the activity of relevant muscles in elite racewalkers. The movements of the pelvic girdle were the most important in terms of optimizing key spatiotemporal variables, which is unsurprising given it articulates with the femur and functions as an origin for many lower limb muscles. Pelvic rotation was associated with longer foot behind ratio and hence stride length ratio, showing that this exaggerated movement in racewalking does compensate to some extent for restrained stride lengths, and should continue to be encouraged by racewalk coaches. As with all bipedal gaits, the arms have a purpose in balancing the lower limb's movements, although there was little evidence to support coaching recommendations that these should be particularly exaggerated. Indeed, elite racewalkers should be mindful of the elbow and shoulder movements used during arm swing: a larger range of shoulder swing movements was found to be associated with lower stride frequency, which could either indicate a shoulder motion that drives a longer stride time, or a lower stride frequency that allows for a longer shoulder swing. In either case, such a movement can be beneficial to some extent but can also lead to visible loss of contact. When taken into consideration with the kinematic data, it is clear that the shoulder muscles mostly absorb energy when racewalking; therefore, completing arm exercises within a training program is encouraged. Other muscle actions, such as those of the abdominal muscles, had few distinct peaks and might therefore function isometrically to restrain excessive thorax movements. Although a comprehensive strength and conditioning program is recommended for racewalkers, coaches should bear in mind that it is an endurance event and resistance to fatigue might be more important than strength.

## Data Availability Statement

The raw data supporting the conclusions of this article will be made available by the authors, without undue reservation.

## Ethics Statement

The studies involving human participants were reviewed and approved by Carnegie School of Sport Research Ethics Committee. The patients/participants provided their written informed consent to participate in this study.

## Author Contributions

HG conducted the data collection and analyses and created the figures and tables. All authors conceptualized and designed the study, wrote the manuscript, edited, critically revised, and approved the final version for submission.

## Conflict of Interest

The authors declare that the research was conducted in the absence of any commercial or financial relationships that could be construed as a potential conflict of interest.
